# Use of Knowledge Transfer Theory to Improve Learning Outcomes of Cognitive and Non-cognitive Skills of University Students: Evidence From Taiwan

**DOI:** 10.3389/fpsyg.2021.583722

**Published:** 2021-03-29

**Authors:** Michael Yao-Ping Peng, Yongjun Feng, Xue Zhao, WeiLoong Chong

**Affiliations:** ^1^School of Economics & Management, Foshan University, Foshan, China; ^2^School of Education, Shaanxi Normal University, Xi'an, China; ^3^School of Marxism, Tangshan Normal University, Tangshan, China; ^4^Department of Education, New Era University College, Kajang, Malaysia

**Keywords:** knowledge transfer, prior knowledge, student learning outcomes, student orientation, higher education

## Abstract

Previous studies have explored a multitude of factors influencing student learning outcomes based on various theories. Knowledge transfer theory was adopted to develop the antecedents of student learning outcomes in the complete learning process. This study aims to explore the conspicuousness between various factors within the structural model, such as knowledge transfer, student orientation, and absorptive capacity, by combining marketing and management concepts with higher education studies. This study takes Taiwanese University students as its research samples, and purposive sampling is adopted. A total of 873 questionnaires are collected in this study. PLS-SEM was used to verify the structural relationship in data analysis via running of SmartPLS. The results indicate that knowledge transfer and student orientation have significant impacts on students' absorptive capacity and learning outcomes and that students' prior knowledge has a positive moderating effect on the relationship between knowledge transfer and absorptive capacities. Based on these findings, the researchers propose feasible suggestions for related issues and future research.

## Introduction

Knowledge economy has been around for dozens of years. So far, it has been applied in social, economic, political, industrial, and military fields, and even in the most significant educational fields. The importance of knowledge is self-evident. In the industrial field, knowledge workers play a crucial part, such that knowledge accomplishments take the place of production factors, including labor and capital from previous economic models (Drucker, 1999). The knowledge accomplishments of knowledge workers mostly lie in higher education because it provides students with the factors required to become a knowledge worker, such as the learning environment, learning guidance, and course teaching opportunities (Morley, 2001; Ding et al., 2016). Thus, taking college students in higher education institutions as the research object, this study discusses the relevance of knowledge acquisition and capacity accumulation.

Under the background of knowledge management, knowledge forms through the conversion process from data to information and then to knowledge. This means that not all received information or data can become knowledge that can be employed by individuals, and a conversion process is required to generate valuable and exclusive knowledge (Dalkir and Beaulieu, 2017). On a theoretical basis, knowledge conversion theory explains the formation of knowledge. The theory focuses on the interaction between tacit knowledge and explicit knowledge (Nonaka, 1994; Nonaka and Takeuchi, 1995; Nonaka et al., 2001). The conversion to valuable knowledge must be carried out on the basis of the prior common knowledge among individuals, which is the prerequisite condition of this theory. Thus, excessive heterogeneity between individuals will lead to a failed process of knowledge conversion. The majority of studies have adopted the knowledge conversion phases asserted by Nonaka and Takeuchi (1995) to discuss the effects of the internal knowledge conversion of enterprises or the knowledge conversion between enterprises on capability and performance. However, few studies have discussed the application of knowledge conversion theory in higher education and conducted empirical analyses of its research framework. Prior traits of students in higher education are highly homogeneous (Kahu, 2013), and curriculum and instruction offered by teachers are examples of knowledge transfer. Thus, the knowledge conversion theory can be applied to the development of college students' capabilities for better learning outcomes.

With the exception of knowledge transfer, teaching style and mode are also some of the determining factors for whether students can obtain knowledge and capabilities from the learning process. The traditional teaching modes can be divided into “teacher-oriented mode” and “student-oriented mode.” These two distinct teaching modes will lead to students taking different learning-development and capability approaches (Sturm and Bogner, 2008). Much like organizations more traditionally associated with marketing strategies and tactics, colleges and universities are gradually moving toward and embracing the marketing concept and a focus on consumer orientation (Bristow and Schneider, 2003; Pesch et al., 2008; Polkinghorne et al., 2017). More and more studies argue that the marketing concept in higher education is favorable for universities to know their own positioning in running schools and provide students with proper teaching and course design (Koris et al., 2015; Polkinghorne et al., 2017) and indicate that University marketers have begun to address related issues, including the assessment of the institution's customer orientation, targeting of market segments appropriate to the strengths of the institution, matching the University's product offerings to the targeted segments' needs, and understanding the complexities of the “customers” (Pesch et al., 2008; Khanna et al., 2014; Koris et al., 2015). In terms of student orientation (SO), teachers give the students the initiative in class, guiding them to make more speeches, break through the limitations of the teaching materials, and adopt diversified teamwork learning modes (Sturm and Bogner, 2008). The provision of an open learning environment is deemed as an intensively student-oriented learning mode (Bauer and Liang, 2003), and the interactive and diversified learning environment is deemed as an important knowledge source. Scholars have argued that an open learning environment allows students to gain better learning outcomes and develop more excellent capabilities (Pesch et al., 2008), but few empirical students have provided a complete integrated structure to verify the significance of SO. Hence, in this study, we have added SO as an antecedent variable of the research framework to explore the correlation between SO and students' capabilities with learning outcomes.

The open learning environment and teachers may or may not help students develop excellent capabilities, although students can obtain valuable information and knowledge from these sources. The knowledge conversion theory points out that individuals can integrate and absorb information and convert it into knowledge. However, the theory seems to have ignored how students use and absorb this valuable knowledge to develop their exclusive capabilities. Zaheer and Bell (2005) in their studies of organizational management combined a resource-based view with network theory, holding that emphasis should be put on the internal capability for absorbing the knowledge of an organization (i.e., absorptive capability) and the capability of an organization for acquiring external knowledge and information (Cohen and Levinthal, 1990). Like the connectedness emphasized by Fabrizio (2009), students should be equipped with approaches and mechanisms to learn, absorb, transfer, and apply knowledge, and to strengthen the benefits presented by knowledge. There are few previous studies in the educational field discussing students' absorption of knowledge. Although enriched and valuable knowledge and information are generated, students' absorptive capability is still a key factor to be measured. Thus, in this study, their absorptive capability is taken as a mediating variable between learning outcomes and knowledge acquisition.

Based on the above statements, this study will make research contributions in the following aspects: (1) applying the knowledge transfer theory to teachers in higher education institutions and discussing its impact on teachers' knowledge transfer; (2) exploring the development and establishment of students' absorptive capability and verifying the relevance of the combination of student capabilities and learning outcomes; and (3) introducing the marketing concept into student learning to discuss the correlation of students' absorptive capability and their orientation to learning outcomes.

## Theory and Hypotheses Development

### Student Absorptive Capacity

Student learning outcomes (SLO) are an important index to judge the effectiveness of student learning. The measurement of learning performance provides a way to know about students' learning status and serves as the basis for improving their learning efficiency and effectiveness (Guay et al., 2008). Studies have shown that learning is a process that promotes the evolution of individual behaviors by virtue of activities or experience, as well as the performance evaluated by a certain evaluation indicator through involvement in curriculum and interaction with teachers and peers during learning (Pike et al., 2011, 2012). Pike et al. (2011) noted that the educational expenditures of higher education institutions and student engagement would affect students' learning performance, so cognitive gains and non-cognitive gains were proposed as variables measuring SLO. Pike et al. (2006) indicated that previous studies tested students' level of learning at different times. In other words, considering students of different grades in a single analysis may result in bias errors in research results. To solve this problem, this study controls the grades of students and reduces the degree of variation in the variables among the samples; moreover, the cognitive gains and non-cognitive gains proposed by Pike et al. (2011) are adopted as indexes for measuring University students' learning outcomes. Cognitive gains means that students' University experience is conducive to general education, quantitative analysis, critical thinking, and writing and speaking. It has significant improvement and progress. Non-cognitive gain tests students' self-knowledge, cooperation, ethical standards, and the response of citizens and community participation (Peng and Chen, 2019; Peng et al., 2019; Li et al., 2020).

Nieto and Quevedo (2005) defined absorptive capacity (AC) as individuals' abilities to identify new values, acquire valuable knowledge, and apply this knowledge to achieve purposes. According to existing knowledge, AC serves to develop and promote new knowledge. Moreover, in the context of higher education, the enhancement of students' capacities and skills will determine how the students utilize, combine, and even fundamentally develop their own core capabilities. Cadiz et al. (2009) indicated that personal AC is the process of applying new knowledge through assessment (identification and filtering of valuable information), assimilation (translating new knowledge into usable knowledge), and application (using knowledge and converting it into usable knowledge). When students have a strong AC, they will be able to produce new insights into the learning process and even enhance the efficiency of teacher knowledge transfer (TKT) in the process of knowledge acquisition to complete the tasks the teacher has explained (Wang and Ahmed, 2007).

Absorptive knowledge is as a crucial part of the process of knowledge conversion, and students need to internalize external new knowledge through the socialization process (Yeoh, 2009). Being embedded in the relationship of mutual benefit and collaboration and trust, students and teachers will enhance the efficiency of communication and knowledge transfer as their interactions increase. Jiang et al. (2008) argued that if students have a good AC, they can improve their capability of utilizing knowledge learned in class, absorb new knowledge more efficiently, and have a deeper understanding of external new knowledge, thus enhancing their professional and general capabilities. In other words, if students are not equipped with AC, they are unable to completely absorb and employ tacit or explicit knowledge transferred from teachers (Chen, 2003). Students with enough AC will conduct open communication and exchange knowledge through common interests and language, and reserve valuable knowledge (Wenger, 1998; Cadiz et al., 2009), which will be beneficial for the improvement of their employability. Nor et al. (2012) indicated that students who are equipped with AC are superior in academic attainment and prior knowledge (PK), and they are able to transfer and employ knowledge efficiently, thus improving their academic performance and employability in the future. Thus, Hypothesis 1 is proposed:

*H1: Student absorptive capacity positively correlates with student learning outcomes*.

### Teacher Knowledge Transfer

According to the cognitive learning perspective, students can leverage their knowledge and resources to build their own capabilities and shape their employability through the use of their abilities. TKT can effectively facilitate students' knowledge to be applied in the management process to create value (Walter et al., 2007). Therefore, in the learning process, students must internalize the information. Knowledge exists in the human mind through learning or experience and then gradually grows with experience, involving personal beliefs, judgments, and value perceptions, in addition to explicit textual behavior, including the implicit mental journey. Polanyi (1962) distinguished between tacit knowledge and explicit knowledge as follows: Explicit knowledge is objective and rational, and it can be encoded and stored in various physical and electronic formats while tacit knowledge is an individual's own experience, reflections, cognitions, or talents, which are difficult to be presented (Astorga-Vargas et al., 2017).

Other scholars have suggested four steps in the process of knowledge transfer: (1) socialization: the beginning of knowledge transfer with the process of tacit knowledge, the facilitation of life experiences, and the capacity among students where they reside and are needed; (2) externalization: this propitiates all activities that are grouped and aimed to facilitate the knowledge management by changing the knowledge from being tacit to being explicit (Inkpen and Dinur, 1998; Nonaka and Von Krogh, 2009; Zhou et al., 2010); (3) combination: there is a process in which different pieces of existing explicit knowledge are merged to create new explicit knowledge; (4) internalization: a process is carried out wherein the student puts into practice what has been learned from their explicit knowledge (Astorga-Vargas et al., 2017). Therefore, based on the above statements, this study defines TKT as a learning process. Teachers will transfer tacit and explicit knowledge to students through various teaching modes, enabling students to integrate it with their own currently held knowledge.

TKT facilitates students' learning more knowledge (Nemanich et al., 2009). As mentioned above, TKT is referred to as a learning process that includes changes in the learning environment, course assignments, and the conversion of the teacher's instruction style. Teachers adopt proper teaching patterns to make students acquire knowledge and curriculum content from visual, auditory, and interactive perceptions, thus improving student learning engagement and learning outcomes via the process of knowledge acquisition and transfer (Weiermann and Meier, 2012). According to the characteristics of TKT, although tacit knowledge is more ambiguous than explicit knowledge, teachers use learning patterns to assist students in acquiring the value of the knowledge. Some scholars indicate that the dynamic between instructor expertise and social richness (that is tacit knowledge transfer) of an in-class environment will make students involved in non-cognitive skills acquisition (Nemanich et al., 2009), including changing original perspectives and providing a deeper insight into issues, etc. (Van Doorn and Van Doorn, 2014). In short, the utilization of explicit knowledge helps to enhance general working abilities and professional working abilities while promoting learning efficiency, such as mnemonics, visual imagery, word associations, and self-regulation learning strategies of forethought, performance, and self-reflection help with learning (Paivio, 2008; Reed, 2013). These explicit knowledge transfer strategies have been found to improve SLO and gain cognitive skills (Van Doorn and Van Doorn, 2014). Besides, explicit knowledge plays an indispensable role in general and professional competence, but the most important aspect of this is its combination with tacit knowledge to increase capabilities of knowledge processing. Moreover, Teigland and Wasko (2009) claimed that TKT will help students to reuse knowledge, solve general problems, interact with teachers, and create new knowledge; the transformation and combination of tacit knowledge is conducive to improving the extent to which students apply knowledge to the real world and have new cognition and insights related to the original issues and matters. In other words, the higher the extent of knowledge transfer, the more capable students are to evaluate and utilize outside knowledge, which is largely a function of the level of prior related knowledge (Tho and Trang, 2015). Based on the above arguments, we propose the following hypotheses:

*H2: Teacher knowledge transfer positively correlates with student learning outcomes*.
*H3: Teacher knowledge transfer positively correlates with student absorptive capacity*.

### Student Orientation

The marketing literature has widely satisfied the topic of why organizations should be customer-oriented and is related to outstanding organizational performance. According to the marketing concept proposed by Deshpande and Webster (1989), the “marketing concept is a specific culture of an organization, enabling the organization to think over strategies and business direction with customers as the center.” In other words, the emphasis of an organization on the functions and operations of marketing orientation signifies the establishment and maintenance of the organizational culture. Some scholars also have proved that marketing experts know well the target customers and can precisely predict their needs and constantly create excellent values for them (Narver and Slater, 1990; Deshpandé et al., 1993).

Many higher education institutions in America have begun to discuss brand value and brand benefits from the perspective of marketing and have established a long-term brand trust since 2001. For instance, the brand of the University of Cincinnati is based on the essence of its brand and the brand's character and attributes, creating a point of difference among competing universities. A total of 130 higher education institutions in the educational system of California are committed to attracting and retaining students. Faculty in universities have begun to employ the marketing concept in academic units and have realized the significance of customer orientation. This means that universities will need to not only satisfy students' needs but also provide students with high-quality education regardless of costs and to consider students as important customers. The concept of SO was thus proposed.

As mentioned above, SO is the application of customer orientation in higher education institutions. Its essence is to build an internal marketing culture, satisfy student needs by treating them as customers, and then create students' values. However, the building and maintenance of University culture is not as easy as modifying the articles of association, but should follow the development of time path and be built from top to bottom and level by level. When higher education institutions feature a high degree of SO, the faculty will be able to provide students with exclusive service-contact experiences and make them feel the institution's care and efforts, which, in turn, encourages the students to succeed academically and to have this achievement as their goal, thus reducing the possibility of suspension or dropping out and increasing student retention (Pesch et al., 2008). Some scholars have asserted that highly student-orientated higher education institutions are helpful to appropriately and rationally adjust students' interest and demands (Olssen and Peters, 2005). To provide favorable educational experiences, such institutions will regard students as customers and listen to and satisfy their needs and desires (Desai et al., 2001). When institutions are capable of transferring educational experience and satisfying students' learning demands, the students can feel the institutions' commitment to satisfying their learning demands (Pesch et al., 2008; Browne, 2010), which will be reflected in the improvement of the quality of the students' learning outcomes. Furthermore, in such circumstances, students are more likely to accept guidance and acquire knowledge and skills from their teachers, thereby enhancing the teaching effectiveness (Koris and Nokelainen, 2015). Thus, Hypotheses 4 and 5 are proposed:

*H4: Student orientation positively correlates with student learning outcomes*.
*H5: Student orientation positively correlates with students absorptive capacity*.

### Prior Knowledge

An individual's explanation of their current situation and information depends on reserves of knowledge and experience they own (Chang and Edwards, 2015). In other words, existing knowledge and experience (that is, PK) will facilitate learners to identify opportunities for learning, but the learners may not know how to draw upon their existed knowledge base in their seeking of knowledge (Ineson et al., 2013; Thompson et al., 2016). The function of PK is to help the learner understand external knowledge and information, and then to combine the intension of acquiring knowledge with their PK (Williams and Lombrozo, 2013; Li et al., 2015), thus generating a more enriched basis of PK (Ineson et al., 2013; Cordova et al., 2014). Therefore, PK is not changeless, but can increase as time goes by, showing a characteristic of path dependency (Williams and Lombrozo, 2013; Li et al., 2015), and the PK can be strengthened based on the learner's learning attitude and motivation (Ineson et al., 2013; Cordova et al., 2014; Hajizadeh and Zali, 2016; Liguori et al., 2019).

Scholars have discussed the effectiveness of PK on the basis of different theories in studies of students' PK. Some empirical studies have indicated that PK has no effect on the improvement of students' learning performance (Müller-Kalthoff and Möller, 2003; Potelle and Rouet, 2003; Mishra and Yadav, 2006), but many scholars still hold that PK is significantly correlated with learning. Based on the cognitive load theory, Amadieu et al. (2009) studied the effect of students' PK on the learning of electronic files and found that students with excellent PK were able to process information and organize their own learning path using their own mental model. Additionally, students with excellent PK were less likely to suffer from learning confusion than were those with poor PK (Jenkins et al., 2003; Mishra and Yadav, 2006). On the other hand, some other studies have shown that PK has no significant effect on subjective learning confusion (Calisir and Gurel, 2003; Müller-Kalthoff and Möller, 2003). The possible reasons may be that the effect is limited to written and explicit knowledge, even if students have rich PK, while in terms of tacit and complex knowledge information, students with rich PK would explore the knowledge connotations more carefully and deeply (Jenkins et al., 2003), helping convert these inquiry processes into AC. Thus, Hypothesis 6 is proposed:

*H6: Students' prior knowledge has a positive effect on the relationship between teacher knowledge transfer and absorptive capacity*.

**Figure 1:**
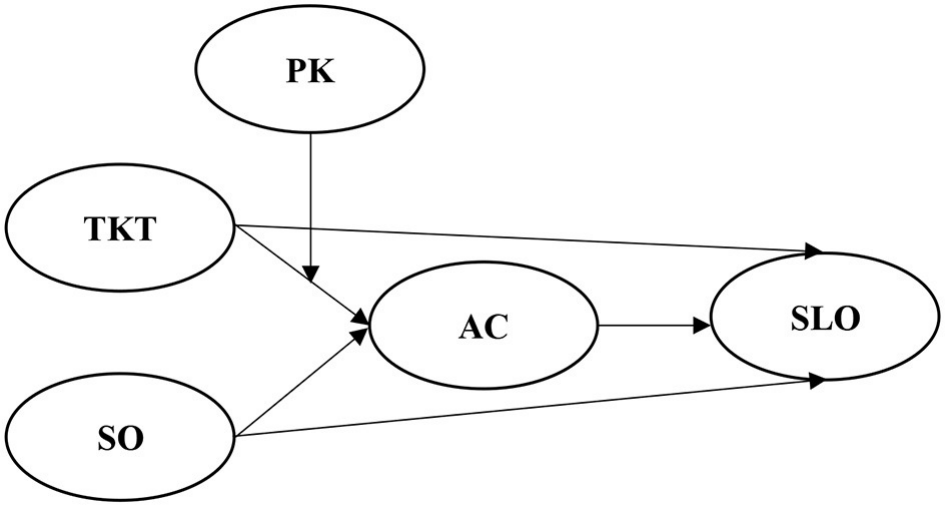
Research framework.

Based on the above hypothesis, this study proposes the following research framework (Figure 1).

## Methodology

### Participants and Sampling

This study involved conducting a questionnaire among University students in Taiwan. Due to the large number of higher education institutions (HEIs) in Taiwan it is difficult to perform tests on all HEIs; therefore, purposive sampling was employed. In addition, to accurately measure college students' perceptions of the variables of the study and enhance external validity, two principles for sampling were set. First, junior and senior students who had adapted to college life were taken as respondents, since freshmen and sophomores may not be able to clearly express their employment intentions, making it impossible to measure the effect of each variable on employability. Second, since HEIs in Taiwan are generally classified into public and private universities, public and private universities account for half of the samples, respectively, to enhance the representativeness of the samples.

The researchers initially contacted colleges and teachers who were willing to complete the questionnaire via telephone and email. Before completing the questionnaires, students were asked to understand the right of attending survey, in order to meet ethical requirements. A total of 1,000 questionnaires were distributed to 18 universities (including nine public and nine private universities). A total of 873 valid questionnaires were received, giving an 87.3% response rate. Among the sample attributes, 532 were from public universities and 341 were from private universities. The study considered University characteristics including institution size, category, and attributes before sampling to increase the generalizability of the study. Regarding the sample structure, 45.8% of respondents were male and 54.2% were female. Most students (76.6%) had not applied for a grant, and the study focused on respondents from the social sciences (56.2% in total, in which Liberal Arts accounts for 12.6%, Management accounts for 21.4%, Law and Politics account for 8.6%, and Education accounts for 13.6%) and the natural sciences (43.8% in total, in which Science accounts for 8.4%, Engineering and Computer Science account for 18.7%, Life Science accounts for 7.2%, Medicine accounts for 3.1%, Bio-resource and Agriculture account for 2.3%, and Technology accounts for 4.1%). This simplified the analysis process and kept the research focused. However, according to different types of HEIs or disciplines, a systematic error may arise and its external validity is questioned and challenged. Several independent sample *t*-tests were used to verify whether the samples of public vs. private universities and social sciences vs. natural sciences differed significantly in terms of research dimensions. The results indicated that the two groups do not significantly differ, so it was deemed appropriate to merge the samples from different universities and disciplines.

### Instrument

To divide SLO into cognitive gains (nine items) and non-cognitive gains (seven items), we adopted the scale proposed by Pike et al. (2011). The scale is based on the characteristics of undergraduates in western countries, such as the UK and the US, and its credibility and validity have been verified; therefore, we found the scale suitable for expansion to the context of Asia. This will also verify the generalizability of the scale and improve its theoretical value for measuring learning outcomes. Following Lee and Tsai (2005) and Cadiz et al. (2009), we measured absorptive capacity in terms of assessment (three items), assimilation (three items), and application (three items). TKT uses the explicit knowledge (five items) and tacit knowledge (four items) scales developed by Zhou et al. (2010). In SO, seven items were selected on the basis of prior scale and item analyses of Asian applications (Pesch et al., 2008). Silva et al. (2013) adopted the construct of student PK in the proposed scale by including 10 items. All items were measured with a five-point Likert scale (1 = totally disagree; 5 = totally agree).

## Results

### Evaluation of the Measurement Model

All scales used in this study were found to be reliable, with Cronbach's α ranging from 0.83 to 0.96. Table 1 shows the reliability of each scale, and the factor loadings for each item therein. In order to gauge validity, this study employed confirmatory factor analysis (CFA) using AMOS 23.0 to verify the construct validity (both convergent and discriminant) of the scales. According to Hair et al. (2010) recommended validity criteria, CFA results show standardized factor loading of higher than 0.5; average variance extracted (AVE) ranges between 0.539 and 0.729; and composite reliability (CR) ranges between 0.800 and 0.918. All three criteria for convergent validity were met, and correlation coefficients were all less than the square root of the AVE within one dimension, suggesting that each dimension in this study had good discriminant validity.

**Table 1:** Measurement properties.

	**1**	**2**	**3**	**4**	**5**	**6**	**7**	**8**	**9**
1. Cognition									
2. Non-Cognition	0.802^**^								
3. Assessment	0.514^**^	0.551^**^							
4. Assimilation	0.498^**^	0.531^**^	0.716^**^						
5. Application	0.521^**^	0.553^**^	0.723^**^	0.777^**^					
6. Explicit	0.533^**^	0.581^**^	0.462^**^	0.518^**^	0.501^**^				
7. Tacit	0.519^**^	0.570^**^	0.429^**^	0.479^**^	0.450^**^	0.849^**^			
8. SO	0.584^**^	0.605^**^	0.652^**^	0.582^**^	0.609^**^	0.415^**^	0.411^**^		
9. PK	0.502^**^	0.588^**^	0.487^**^	0.382^**^	0.428^**^	0.529^**^	0.567^**^	0.474^**^	
Mean	3.579	3.635	3.580	3.776	3.691	3.933	3.893	3.436	3.437
SD	0.646	0.667	0.681	0.651	0.687	0.663	0.689	0.672	0.776
α	0.914	0.908	0.864	0.818	0.867	0.927	0/908	0.946	0.935
AVE	0.595	0.646	0.787	0.733	0.790	0.774	0.785	0.755	0.633
CR	0.929	0.927	0.917	0.892	0.919	0.945	0.936	0.956	0.945

** * if p < 0.01*.

### Testing Structural Model Fit

Before proceeding to examine the structural model, we first tested model fit. Henseler et al. (2015) proposed three model fitting parameters: the standardized root mean square residual (SRMR), the normed fit index (NFI), and the exact model fit. According to Henseler et al. (2015), the evaluation standards for convergent validity are as follows: (1) NFI should be larger than 0.9, (2) SRMR should be <0.08, and (3) exact model fit, which tests the statistical (bootstrap-based) inference of the discrepancy between the empirical covariance matrix and the covariance matrix implied by the composite factor model. Dijkstra and Henseler (2015) suggested the *d_LS* (squared Euclidean distance) and *d_G* (geodesic distance) as two different ways to compute this discrepancy. Henseler et al. (2015) indicated that *d*_*ULS*_ and *d*_*G*_ < the 95% bootstrapped quantile (HI 95% of *d*_*ULS*_ and HI 95% of *d*_*G*_).

In this study, the SRMR value was 0.055 (<0.08) and the NFI was 0.912 (>0.90) and the *d*_*ULS*_ < bootstrapped HI 95% of *d*_*ULS*_ and *d*_*G*_ < bootstrapped HI 95% of *d*_*G*_ indicating that the data fit the model well.

### Inner Model Analysis

To assess the structural model, Hair et al. (2017) suggested looking at the *R*^2^, beta (β), and the corresponding *t*-values via a bootstrapping procedure with a resample of 5,000. They also suggested that in addition to these basic measures, researchers should also report the predictive relevance (*Q*^2^) as well as the effect sizes (*f* ^2^). As asserted by Sullivan and Feinn (2012), while a *p*-value can inform the reader whether an effect exists, it will not reveal the size of the effect. In reporting and interpreting studies, both the substantive significance (effect size) and statistical significance (*p*-value) are essential results to be reported (*p* = 0.279). Prior to hypotheses testing, the values of the variance inflation factor (VIF) were determined. The VIF values were <5, ranging from 1.413 to 1.930. Thus, there were no multicollinearity problems among the predictor latent variables (Hair et al., 2017).

**Figure 2:**
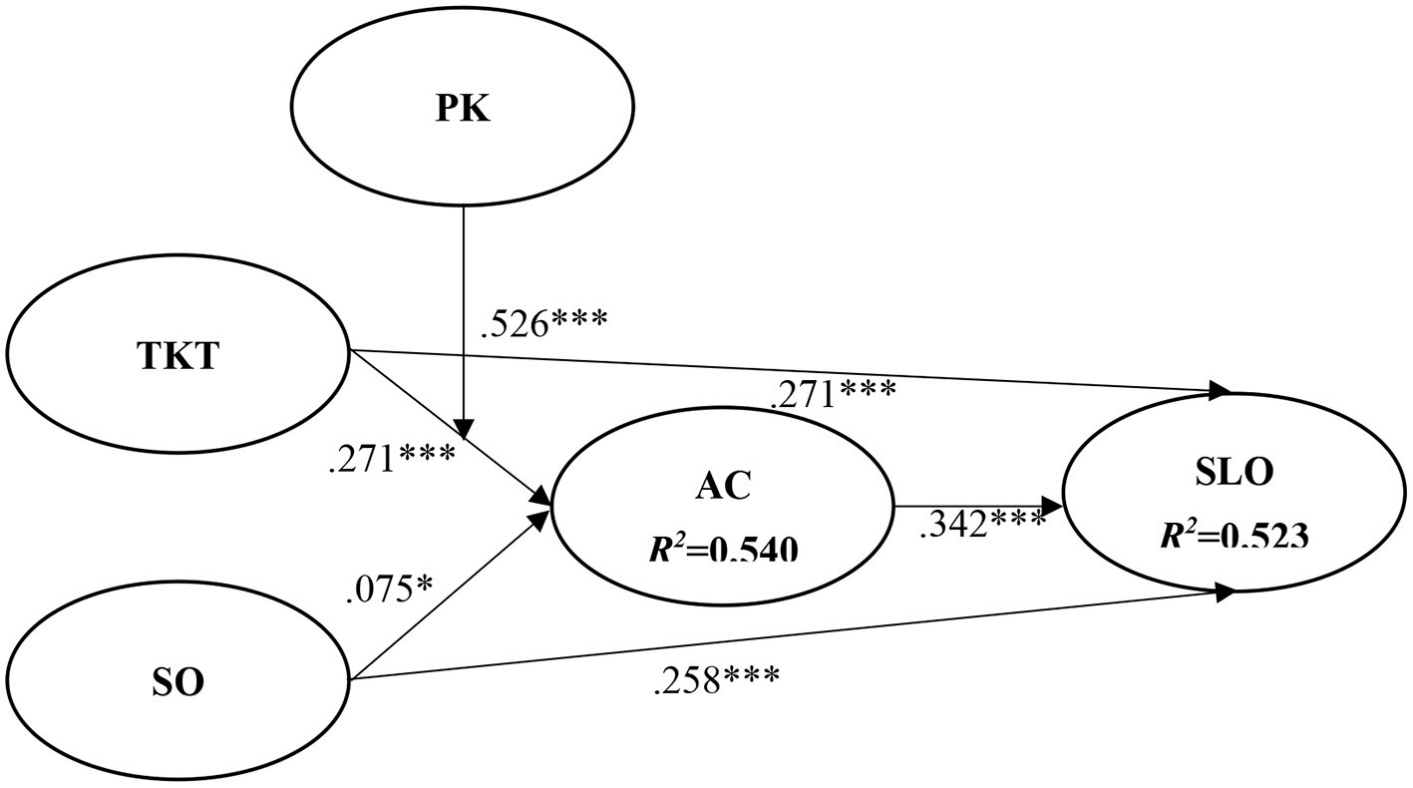
Path coefficients of structural model. * if *p* < 0.05; *** if *p* < 0.001.

Figure 2 and Table 2 show the results of the hypothesized relationships and standardized coefficients in the inner model. The results showed that student absorptive capacity (β = 0.342, *p* < 0.001) was positively and significantly related to SLO, supporting H1. Moreover, TKT (β = 0.271, *p* < 0.001) and SO (β = 0.258, *p* < 0.001) were positively and significantly related to SLO, supporting H2 and H4. Similarly, TKT (β = 0.271, *p* < 0.001) and SO (β = 0.075, *p* < 0.05) were positively and significantly related to student absorptive capacity, supporting H3 and H5. In addition, our results found that moderating effect of student PK (β = 0.562, *p* < 0.001) was positively and significantly supporting H6. The Stone–Geisser *Q*^2^ values obtained through the blindfolding procedures for student absorptive capacity (*Q*^2^ = 0.421) and SLO (*Q*^2^ = 0.447) were larger than zero, supporting the idea that the model has predictive relevance (Hair et al., 2017).

**Table 2:** Results of the hypotheses testing.

**Paths**	**Std. β**	**Std. error**	***t* value**	**Decision**	**Significance**	**VIF**	***f*^**2**^**
					**CI (2.50%−97.5%)**		
H1: AC → SLO	0.342	0.038	8.993	Support	CI (0.274–0.425)	1.652	0.229
H2: TKT → SLO	0.271	0.035	7.705	Support	CI (0.203–0.342)	1.930	0.104
H3: TKT → AC	0.271	0.036	7.593	Support	CI (0.201–0.342)	1.692	0.144
H4: SO → SLO	0.258	0.041	6.323	Support	CI (0.172–0.330)	1.704	0.098
H5: SO → AC	0.075	0.039	1.930	Support	CI (0.002–0.157)	1.774	0.003
H6: Moderating effect of PK	0.526	0.035	14.892	Support	CI (0.454–0.594)	1.413	0.647

*CI, confidence intervals (Lower bound–Upper bound)*.

## Conclusions

### Discussion

In the process of verifying the relationship between TKT and students' learning, we obtained a result that the knowledge transfer theory has a significant effect on explaining the training and development of student capability, which is identical to the findings of Jiang et al. (2008) and of Yeoh (2009). Most previous theories about knowledge transfer have discussed the processing and role of organizations and enterprises in knowledge management (Inkpen and Dinur, 1998; Nonaka and Von Krogh, 2009; Zhou et al., 2010). In this study, we developed the effect of knowledge transfer on SLO on the basis of teachers and verified the importance of its existence (Sadler, 2010). This shows the compatibility of management theory in the context of higher education, which fully demonstrates the diversified development of cross-field research and also verifies the feasibility of interdisciplinary research.

In addition to knowledge transfer theory, this study has also verified the necessity of applying the marketing concept proposed by Deshpande and Webster (1989) in higher education institutions. Based on the view of customer orientation, higher education institutions build the student-oriented culture, making administrative and academic units focus on student learning and providing services that satisfy student demands, thus improving the learning environment and teaching contents and enhancing students' capability. The results of this study are also identical with the statements of Cho (2012) and Blömeke (2012). Specifically, driven by SO, teachers will adopt effective teaching strategies and provide knowledge and feedback required by students so as to develop students' absorptive capacity and strengthen the basis of the students' learning outcomes.

As deduced by this study, student absorptive capacity is positively correlated with learning outcomes. However, the research result is exactly contrary to the deduction, which is different from Sadler's (2010) argument. Sadler (2010) asserted that the development of student capability is a dynamic and complex process in which students' coursework quality, their absorption of tacit knowledge and know-how, the feedback from their teachers (Sadler, 2009), and the quality of their professional knowledge learning are covered, shaping the capabilities that students are supposed to be equipped with. It can be seen from this that there is a circular reasoning of tautology between absorptive capacity and learning outcomes, but the cause and effect remain to be verified. This study proves that the absorptive capacity has no correlation with learning outcomes and encourages future researchers to discuss the relationship of the two items from a different perspective. Absorptive capacity has no mediating effect in the process of knowledge transfer, which is distinct from the framework model of “knowledge source acquisition → capability building → outcome reveal” built in this study, and also disagrees with the opinions of Jiang et al. (2008). The possible reason may be that there are cognitive differences between individual enterprises and individual students in terms of capacity and outcome. Some scholars have expressed the belief that capability is positively correlated with performance in the context of enterprises (Cohen and Levinthal, 1990; Nieto and Quevedo, 2005), but few studies have clarified the differences between capability and outcome in the context of student learning.

Finally, the research results on the moderating effect of student PK support the opinions of Jenkins et al. (2003), Mishra and Yadav (2006), and Franck et al. (2008). Students need to be equipped with an appropriate knowledge base to understand and absorb concepts and views proposed by teachers in their courses. However, Seery (2009) argued that the differences in gender and disciplines should also be considered while discussing prior capacity, because statistically more women students always perform better in reading skills and more men students perform better in scientific knowledge (O'Reilly and McNamara, 2007). Thus, different results may be obtained if varied factors are considered in the future to discuss the influence of PK.

### Implications

Teachers provide students with deep and diversified counseling services, as well as explicit professional knowledge. Students' learning demands change with time. Although students will reflect their demands on instructional assessments, teachers can only use the instructional assessments as a reference for adjusting their teaching styles. This study suggests that the transmission of explicit or tacit knowledge needs effective and clear teaching and course design. In particular, tacit knowledge needs to be presented by the design of knowledge externalization. Thus, teachers should increase the number of discussions over practices and cases and *guide* students to think and solve problems. Learning by doing can enhance students' knowledge absorption and transfer.

The assessment of student-oriented courses should put emphasis on the dynamic learning process and focus on the interaction between students and their own problem-solving abilities. Students can benefit, internally and externally, from the strengthening of SO. Internally, universities apply marketing concept to establishing a strong student-oriented culture, so as to make students feel that the University attaches great importance to student learning and lead students to take the initiative to adopt hardware tools and learning environment for achieving learning targets. Similarly, when student learning autonomy and initiative are higher, the interaction between faculties and students can be enhanced; thus, schools can both have a clearer comprehension of students' learning needs and specifically plan curriculum design and hardware and software facilities that are conducive to cultivating absorptive capacity for students. Externally, applying the marketing concept to the daily tasks of universities' administrative and academic units will help build the word of mouth among students and set up universities' long-term brand equity and reputation, thus enhancing the positive recognition of external persons of interest. The SO culture should be implemented level by level from top to bottom, and the University management should focus on understanding students' learning demands, increase communication with students, and narrow the communication gap between universities and their students. Therefore, this study suggests that the University management interacts and communicates with student representatives several times each semester. Universities should reflect on and take corrective measures for their shortcomings based on the feedback of student representatives and provide necessary learning conditions under reasonable and flexible conditions. Additionally, universities should conduct satisfaction surveys among students at set intervals and adjust the decision-making process and task executions based on the results of the surveys. For instance, universities might adjust the collection of books in the library and increase the number of self-study spaces.

Students in the same class have distinct prior capabilities from each other, and TKT and educational experience are also different, so the homogeneity of students' prior capability should be considered first in teaching practices. Therefore, this study suggests that universities use course grading and diversion as the outpost of distinguishing students' prior capabilities. The information about the results of course grading and diversion is provided for class or course tutors. At the start of professional courses, class tutors should provide counseling services for students, expecting to realize the homogeneity of the knowledge bases among the students and to reach the effect that birds of a feather flock together. Students are able to state their own views in discussion stage in class, instead of not knowing what to do.

### Limitations and Future Research Directions

This study has its contributions to knowledge transfer theory, SO, and SLO. However, some limitations still exist and need to be improved in further studies. First of all, although great achievements have been made to knowledge transfer in the field of business management, few studies have discussed the effect of teacher's knowledge transfer on absorptive capacity and learning outcomes under the background of higher education. This study builds the antecedent dimension of absorptive capacity and learning outcome using the knowledge transfer theory (teacher's knowledge transfer and SO) and also puts forward important findings for learning theories. However, many other theories are also suitable for explaining how to enhance students' learning capabilities, and the outcomes of these theories were not part of this study, such as the need-hierarchy theory (Milheim, 2012), the social cognitive theory (Van Gog and Rummel, 2010), and the self-regulation theory (Cohen, 2012). Thus, we suggest that future studies build the dimensions of the sources of knowledge that affect SLO in different theoretical models.

Furthermore, knowledge transfer theory contains the interaction pattern between knowledge providers and knowledge receivers. In this study, only students were sampled, and the effect brought by knowledge transfer and the influence on subsequent variables have been explored from the aspect of students, but the knowledge providers (i.e., teachers) have not been surveyed. In this regard, the study suggests that subsequent researchers can add teachers in the questionnaire to conduct cross-level hierarchical model analysis, so as to know the actual interactive conditions and situations between teachers and students and then enrich the significance of practice.

However, these research findings do show that SO and absorptive capacity have no positive correlation with SLO, which is inconsistent with the deduction of this study. The possible reasons may be that the impact of other factors has been ignored, in addition to the adaptability of the research tools mentioned above. In the research framework of this study, the correlation of SO and absorptive capacity with learning outcomes is discussed, combined with the marketing concept, which may result in relevant interferential or mediating variables. Thus, we suggest that future researchers consider relevant interferences or mediating variables while discussing the path of knowledge source → learning capability → learning outcome, thereby enhancing the reasonableness of student learning theories.

Moreover, since students' actual academic attainment of data is private and not accessible, the respondents are required to fill in the questionnaire scale designed in this study. However, memories may deviate from the actual situation, and thus, the correlation of knowledge source with learning capabilities would be clearer if students' actual academic attainment of data could be collected while taking into account research ethics. Besides, in this study, despite the fact that there is no difference in all the variables between social and natural disciplines, previous studies have indicated that different disciplines and students' genders result in different research outcomes; thus, it is necessary to incorporate disciplines and genders into issues related to student learning. As the study aims to strengthen the degree of theoretical generalization, the influence that gender and discipline bring to the research model is not discussed in the research findings. As a consequence, it is suggested that subsequent researchers can add the variable of students' background for comparative analysis to provide more valuable insights and enrich theoretical connotations.

## Data Availability Statement

The raw data supporting the conclusions of this article will be made available by the authors, without undue reservation.

## Ethics Statement

The studies involving human participants were reviewed and approved by Ethics Committee in University of Taipei. The patients/participants provided their written informed consent to participate in this study.

## Author Contributions

This study is a joint work of all authors. MY-PP, YF, WC, and XZ contributed to the ideas of educational research, collection of data, and empirical analysis. MY-PP and YF contributed to the data analysis, design of research methods, and tables. MY-PP, XZ, and YF participated in developing a research design, writing, and interpreting the analysis. All authors contributed to the literature review and conclusions.

## Conflict of Interest

The authors declare that the research was conducted in the absence of any commercial or financial relationships that could be construed as a potential conflict of interest.
